# Individual responses to purple grape juice consumption on endurance, explosive power, and fatigue in young male elite soccer players

**DOI:** 10.3389/fnut.2025.1559917

**Published:** 2025-06-13

**Authors:** Alireza Niknam, Kamran Tahmasebi, Mohammad Hemmatinafar, Walaa Jumah Alkasasbeh, Mark E. T. Willems, Maryam Koushkie Jahromi, Morteza Bagheri Kalayeh, Mohammad Hadi Zare

**Affiliations:** ^1^Department of Sport Science, Faculty of Education and Psychology, Shiraz University, Shiraz, Iran; ^2^Department of Exercise Physiology, Faculty of Sport Sciences, University of Isfahan, Isfahan, Iran; ^3^Department of Administration and Curriculum, Program of Sports Management and Training, Faculty of Arts and Educational Sciences, Middle East University, Amman, Jordan; ^4^Department of Physical Education, Faculty of Sport Science, The University of Jordan, Amman, Jordan; ^5^Institute of Applied Sciences, University of Chichester, Chichester, United Kingdom; ^6^Department of Exercise Physiology, Faculty of Sport Sciences and Health, University of Tehran, Tehran, Iran

**Keywords:** carbohydrate, endurance performance, grape juice, jump performance, soccer players

## Abstract

**Background:**

Purple grape juice (PGJ), a natural carbohydrate- and polyphenol-rich supplement, may enhance exercise performance. This study, incorporating individual response analysis, examined the acute effects of PGJ ingestion on endurance, explosive power, and perceived fatigue in elite male soccer players.

**Methodology:**

Twenty-two U-20 male soccer players [Age: 19.7 ± 0.3; height: 178 ± 4 cm; body mass: 72 ± 5 kg; body mass index (BMI): 22.6 ± 0.9 kg/m^2^] participated in an equally allocated, double-blind, crossover design study. Participants were allocated to one of two conditions: (1) purple grape juice (PGJ, *n* = 11) or (2) placebo (PLA, *n* = 11). The PGJ or placebo was ingested in four equal portions (10 ml/kg of body mass PGJ or PLA diluted with water) starting 4 h before the test and continuing every hour, with the final ingestion 60 min before the 30-15 Intermittent Fitness Test (IFT). The placebo consisted of a calorie-free, grape-flavored liquid designed to match the taste and appearance of the PGJ. A 7 days washout period was maintained between the two conditions. Perceived fatigue and standing long jump (SLJ) performance were assessed at baseline, immediately and 5 min after the IFT. Maximum oxygen consumption (VO_2*max*_), time to exhaustion (TTE), and final velocity in the IFT (VIFT) were recorded during the test. The smallest worthwhile change (SWC) analysis was used to evaluate individual responses.

**Results:**

Purple grape juice improved VIFT (*P* < 0.001, Cohen’s d = 0.58), TTE (*P* < 0.001, Cohen’s d = 0.69), and VO_2*max*_ (*P* < 0.001, Cohen’s d = 0.56) compared to PLA. No effects were found for SLJ or perceived fatigue (*P* > 0.05), SWC analysis revealed that 77% of participants showed improvements in VIFT and VO_2*max*_, and 54% showed improvements in SLJ 5 min post-IFT.

**Conclusion:**

Purple grape juice supplementation enhanced endurance performance parameters in most participants, with significant individual variability in response. These findings highlight the potential benefits of PGJ, particularly for athletes more responsive to its effects, emphasizing the need for personalized supplementation strategies.

## Introduction

Soccer players’ physical performance is one of the critical factors that can affect the success of soccer teams (1–3). Although various physical parameters play a role in the optimal performance of soccer players, previous research has shown that aerobic endurance and anaerobic power are essential (2, 4–7). Soccer players cover approximately 10–13 km per game, which includes intervals of walking, running, and sprinting (8, 9). In addition, soccer players are required to execute various physical tasks, including jumps, tackles, and kicks, which demand strength and power. The nature of these activities is contingent upon the player’s position and the prevailing game conditions (10, 11). Therefore, improving indicators related to aerobic endurance and anaerobic power can increase team and individual success.

Although soccer players’ training and conditioning can influence their aerobic endurance and anaerobic power, nutritional strategies significantly affect their physical, cognitive, and technical performance, both in the short and long term (12, 13). In this regard, previous studies have shown that the ingestion of carbohydrates near and during the match improves the endurance performance of soccer players (12–15). However, carbohydrate sources include a wide variety of foods, powders, jellies, juices, and syrups, and their impact on athletes’ performance requires further studies (16). Recently, sports nutrition studies have focused on natural fruit juices rich in carbohydrates and polyphenols (16–18). Compared to other supplements, grape juice is cheaper, contains antioxidants and carbohydrates, and is free of World Anti-Doping Agency (WADA) banned substances (19, 20). Grape juice contains water, a high concentration of sugars, organic acids, minerals, phenolic compounds, and other nutrients such as vitamins, proteins, fatty acids, and amino acids (21–23). Carbohydrates are found in the form of fructose and glucose (17, 21). Phenolic compounds are the most abundant compounds, followed by sugars and acids (21). Anthocyanins are the main phenolic compounds in red/purple grape juice, while flavan-3-ols are more abundant in white grape juice (21). In addition, grape juice can be used as a natural isotonic drink to supply electrolytes and hydrate the body during exercise (21).

Therefore, grape juice is one of the exciting candidates that has shown promising results in some sports nutrition interventions. For example, an animal study showed that purple grape juice (PGJ) protected against oxidative damage caused by exhausting exercise in different tissues (24). These antioxidant effects and improved endurance performance have also been confirmed in several human studies (19, 25–28). In a study by de Sousa et al. (29), it was demonstrated that the administration of PGJ (10 ml/kg body mass, 2 h prior to the test) led to a significant enhancement in the time to exhaustion during endurance exercise, conducted at a velocity corresponding to 80% VO_2*max*_, among recreational runners (29). Similar results were also reported by de Lima Tavares Toscano et al. (25). Indeed, de Lima Tavares Toscano et al. (25) showed that single-dose ingestion of PGJ (10 ml/BM) has an ergogenic effect on recreational runners by increasing running time to exhaustion and increasing antioxidant activity (25). However, a meta-analysis of seven studies reported that grape juice had no significant effect on physical performance (30). However, the number of analyzed studies was low, especially in the athlete population (only three), which may affect the findings (30).

Given the increasing scientific and practical interest in natural, polyphenol-rich supplements, alongside the growing demand for evidence-based strategies to enhance performance in elite athletes, this study sought to fill a gap in the literature. PGJ has shown promising antioxidant and ergogenic effects in recreational populations; its acute impact on the physical performance of elite-level soccer players remains largely unexplored. This gap is critical considering the complex physiological demands of soccer, which combines sustained aerobic activity with repeated bouts of high-intensity anaerobic efforts. Therefore, the present study aimed to investigate the acute effects of PGJ supplementation on aerobic and anaerobic performance outcomes for the first time in elite male soccer players. To address this gap, the present study investigated the acute effects of PGJ supplementation in elite male soccer players and applied the smallest worthwhile change (SWC) approach—a novel analytical method—to better capture practically meaningful changes in performance outcomes.

## Materials and methods

### Participants

Twenty-two elite young male soccer players (U-20 professional players from Zob Ahan Esfahan F.C.), with an average of 3.14 ± 0.94 years of experience in the Iran Soccer League, (U-20 professional soccer players (Zob Ahan Esfahan F.C); Age: 19.7 ± 0.3 years; height: 178 ± 4 cm; body mass: 72 ± 5 kg; body mass index [BMI]: 22.6 ± 0.9 kg/m^2^) volunteered to participate in this study. The inclusion criteria included participation in official soccer competitions in the past 3 years, being physically healthy with no injuries, having no history of allergy to grapes, and having no history of smoking. Furthermore, exclusion criteria included contracting an infectious disease during the study (e.g., cold or flu), severe musculoskeletal injuries, and taking drugs or ergogenic supplements 2 weeks before the start of the trial. Before the implementation of the intervention, the study procedures were explained to the participants, and written consent was obtained. This study was reviewed and approved by the Ethics Committee of the Shiraz University, Shiraz, Iran, and carried out under the Declaration of Helsinki (ID: IR.US.PSYEDU.REC.1403.085).

### Sample size calculation

The number of participants in this study was determined based on the study by de Lima Tavares et al. (25), according to which a single dose of purple grape juice led to a significant improvement in antioxidant activity (effect size = 1.9) and time to exhaustion (effect size = 0.32) compared to placebo. Using G*Power 3.1, considering the confidence interval of 95% and the analysis power of 0.80. Based on the effect size of antioxidant capacity, it was determined that a minimum of 10 participants would be required for the study.

### Study design

**FIGURE 1:**
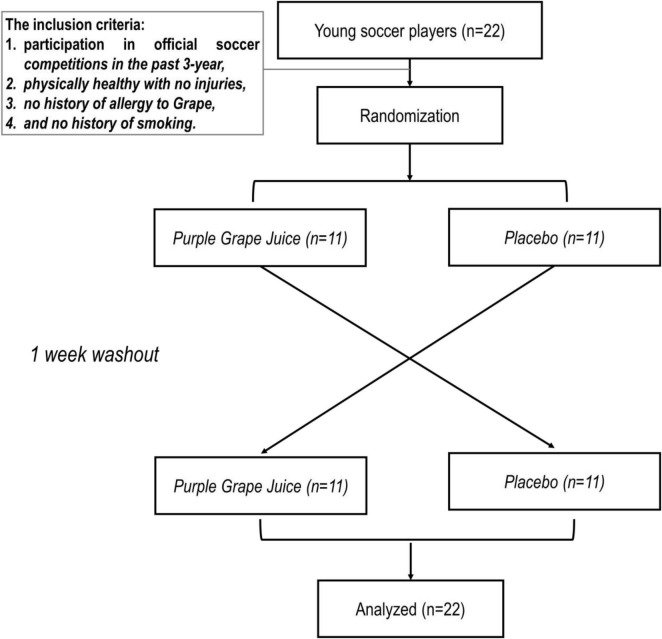
Allocation of participants and crossover study design.

**FIGURE 2:**
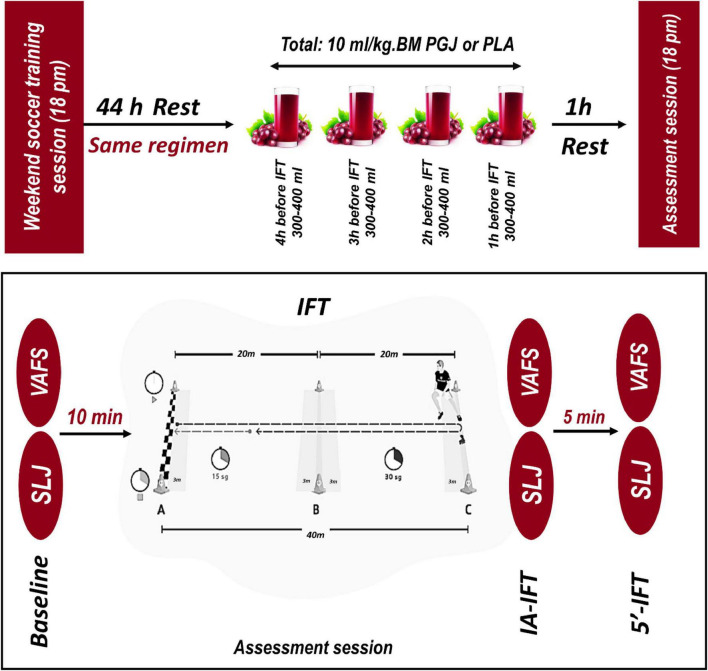
Supplementation protocol and functional tests in the study. PGJ, purple grape juice; PLA, placebo; BM, body mass; VAFS, visual analog fatigue scale; SLJ, standing long jump; IFT, 30-15 Intermittent Fitness Test; 0’-IFT, Immediately after IFT; 5’-IFT, 5 min after IFT.

This study was carried out in an equally allocated, cross-over, placebo-controlled, and double-blinded manner (Figure 1). Before the beginning of the investigation, the participants experienced a familiarization session. During this session, participants were familiarized with all testing protocols and methods. Subjects participated in two separate test sessions. In each test session, the participants were randomly placed in one of the two conditions, including 1- Purple Grape Juice ingestion (PGJ, *n* = 11) and 2- Placebo (PLA, *n* = 11). A 1 week interval was considered a washout period for each condition (Figure 1). The PGJ or placebo was administered in four equal doses (10 ml/kg of body weight PGJ or PLA diluted with water), commencing 4 h before the test and then every hour after that, with the final dose taken 60 min before the 30–15 Intermittent Fitness Test (IFT) (Figure 2). It has been established in earlier research that a 10 ml/kg dose of grape juice positively influences endurance performance (25, 26). Each test session measured perceived fatigue by Visual Analog Scale (VAFS), Standing long jump (SLJ), and IFT. VAFS and SLJ were measured at rest/baseline, immediately after the IFT (0’ post-IFT) and 5 min after the IFT (5’post-IFT) (Figure 2). All trials were completed at the same time (between 5.30 and 6.30 p.m.). To control the diet, the participants’ main meals (breakfast: 8.30 a.m., lunch: 12.30 p.m., and dinner: 7.30 p.m.) were served in the self-service restaurant of the club starting 48 h before the IFT. Snacks were delivered in packs to each participant, and they were asked to consume according to the instructions. Furthermore, the contribution of macronutrients in participants’ diets was the same (5 g carbohydrates/kg body mass, 1.7 g protein/kg body mass, and 1 g fat/kg body mass). Participants were instructed to maintain their diet during the test period and avoid vigorous exercise for 48 h before each test session.

Participants were provided with a list of common dietary sources of caffeine (e.g., coffee, tea, chocolate, and energy drinks) and were instructed to abstain from consuming them for 48 h prior to each exercise test session, in order to minimize potential confounding effects of caffeine intake on performance-related outcomes.

In addition, the players were prohibited from consuming any nutritional supplements 2 weeks before the start of the trial.

### Purple grape juice and placebo supplementation

Participants in the study were provided with either PGJ, derived from the Sahebi purple grape variety cultivated in Isfahan Farms, Iran, or a PLA. To ensure blinding, the placebo was formulated to resemble the taste and appearance of the PGJ closely. The placebo contained water, a low-calorie sweetener powder (containing isomalt, maltodextrin, and sucralose, Kamvar, Iran), grape flavoring (Niko Shimi, Iran), and natural beetroot red food coloring (Freer, Iran).

To ensure the consistency and suitability of the supplementation protocol, the PGJ was diluted with water at a ratio of 120 ml water per 100 ml of juice, resulting in a 7% carbohydrate solution. This specific dilution was applied to standardize carbohydrate intake and reduce the natural sugar concentration, thereby minimizing the risk of gastrointestinal distress during exercise—a common issue with higher sugar-content beverages. The PLA was designed to match the PGJ in volume, color, and taste. This PLA formulation enabled accurate blinding, ensuring participants and researchers remained unaware of the conditions. The composition of the PGJ and PLA solutions, including their macronutrient profiles and ingredient breakdown, are detailed in Table 1. By controlling for these variables, the study aimed to isolate the effects of grape juice ingestion on performance outcomes without the confounding influence of sensory cues or metabolic differences induced by non-standardized beverages.

**TABLE 1:** Nutritional characteristics of placebo and purple grape juice per 100 ml.

Nutrients	PGJ	PLA
Calories (kcal)	65	21
Carbohydrate (g)	16.2	4
Of which sugars (g)	15.5	0.12
Fructose (g)	8.7	–
Glucose (g)	6.5	–
Sucrose	∼0.3	–
Fiber (g)	0.7	–
Protein (g)	0.5	0
Fat (g)	Trace	0
Calcium (mg)	10	–
Iron (mg)	Trace	–
Potassium (mg)	102	–
Sodium (mg)	5	1.32
Magnesium (mg)	10	–
Vitamin C (mg)	Trace	–
Niacin (mg)	Trace	–
Vitamin E (mg)	Trace	–
Riboflavin (mg)	Trace	–
Pyridoxine (mg)	Trace	–
Thiamine (mg)	Trace	–
Anthocyanin (mg)	40	–

PGJ, purple grape juice; PLA, placebo.

Each participant was provided with four individually labeled bottles, each containing 300–400 ml of the assigned drink. It is important to clarify that these drinks were not composed solely of grape juice. To reduce potential gastrointestinal discomfort, we diluted the grape juice by mixing 100 ml of grape juice with 120 ml of water (i.e., a 1:1.2 dilution ratio). The intended dosage of pure grape juice was 10 ml per kg of body mass. For example, for a participant weighing 70 kg, the required dose of grape juice would be 700 ml. After dilution (with 120% water), the total volume becomes 700 + 840 = 1,540 ml, which was then divided across the four bottles (approximately 385 ml per bottle). This explains why each bottle contained 300–400 ml — a substantial portion of which was just water used for dilution purposes. This clarification ensures that the actual intake of grape juice (and its bioactive compounds) was carefully calculated based on body mass, while the increased total volume was due to the necessary dilution for participant comfort. Clear instructions for timing and consumption were provided to ensure consistency. The first bottle was consumed 4 h before the test, followed by one bottle every hour, with the final bottle ingested 60 min before the IFT (Figure 2). This dosing protocol ensured that the total volume consumed was proportional to body mass while maintaining a consistent timing schedule for all participants.

### Functional tests

Intermittent Fitness Test is designed to assess the aerobic capacity of soccer players (31). The 30-15 IFT consists of 30 s shuttle runs interspersed with 15 s walking recovery periods. The test’s starting speed is 8 km/h (i.e., the first 30 s shuttle run), increasing by 0.5 km/h for every 30 s stage after that. Individuals must run back and forth between the two lines set 40 m apart at a speed dictated by an audio “beep” (Figure 2). As the individual progresses through the levels, the time between the beeps decreases, giving the individual less time to complete each shuttle, thus increasing the speed/intensity of the test. The two 3 m zones in the middle of the testing area (6 m) allow gauging of the required running speed and adjusting their speed accordingly (i.e., speed-up or slow-down). The two 3 m end zones/turning lines also help guide the individual to adapt and maintain their speed. During the 15 s recovery period, individuals must walk forward toward the closest 3 m zone, where they will start the next running stage. Individuals must consistently reach the next 3 m zone – either the middle or end zone. Failure to reach the next 3 m zone on three consecutive occasions results in elimination from the test. The speed of each participant’s last stage was recorded as the test score (VIFT: velocity–intermittent fitness test). Maximum oxygen consumption (VO_2*max*_) was also calculated based on VIFT using equation 1 (31).


VO(ml⋅kg⋅-1min)-12m⁢a⁢x=28.3-(2.15×G)-(0.741×

A)-(0.0357×W)+(0.0586×A×VIFT)+

(1.03×V⁢I⁢F⁢T)

VIFT: the final velocity–intermittent fitness test (km/h), G refers to gender (male = 1; female = 2), A: age (in years), W: weight (in kilograms).

The SLJ test measured the maximum horizontal jump ability. For this purpose, the participants were asked to stand behind the marked line on the ground and jump with both legs as far as possible allowing swinging of the arms. Participants were asked to land on both feet, and the distance (cm) between the heel contact point and the starting line was measured. Each participant was allowed three attempts. The best of three attempts was taken for analysis (32, 33).

Perceived fatigue was measured using a visual analog fatigue scale (VAFS) (34, 35). A 10 cm linear image was provided on paper to the participants. “No Fatigue” was written at one end of the line, while “Maximum Fatigue” was portrayed at the opposite end. Participants determined their perception of the intensity of Perceived fatigue with a cross mark “x” on the lines before, Immediately, and 5 min after IFT. The amount of perceived fatigue for each participant was recorded in millimeters with a ruler by measuring the distance from the origin of the line to the marked “x” (35–37). Participants were familiarized with the proper use of the Visual Analog Scale (VAS) before the experiment.

### Nutritional control

Forty-eight hours prior to the assessment sessions, participants at Zob Ahan Esfahan F.C. were provided with three main meals (breakfast at 8:30 a.m., lunch at 1 p.m., and dinner at 7:30 p.m.) in the self-service restaurant. Additionally, each participant received standardized packs containing identical snacks [consisting of 1 medium banana, ten pistachios, and a protein bar (Karen, Iran)] and was given specific consumption guidelines. Nutrient analysis was conducted using Diet Organizer version 3 software.

### Training protocol

To maintain ecological validity and replicate real-world conditions under which elite athletes typically continue their regular training during supplementation, participants were instructed to keep their routine team training throughout the testing period. This also helped avoid potential detraining effects that could confound performance outcomes.

All participants were members of the same training camp, and their training regime was the same under the supervision of trainers. All subjects participated in the following training program: five training sessions of 90 min per week, including 10 min of warm-up, 20 min of physical training (core stability, speed, agility, and quickness), 10 min of technical training, 20 min of tactical training, 2 min of the training game, and at the end, there was cooling for 5 min. Strength and power training occurred once per week as part of team training. It consisted of a combination of plyometric (single leg hops, drop jumps, box jumps, squat jumps: 3 sets × 8 repetitions for each) and resistance exercises (3–4 sets, 10–12 repetitions, 75–80% of a maximum repetition). The training program was similar for all participants. Also, 1 day a week was dedicated to friendly matches, in which each participant played for approximately 40–45 min.

### Data analysis

The data underwent analysis using both descriptive and inferential statistical methods. Means and Standard deviations (SDs) were calculated for all the dependent variables. Values are expressed as mean ± SD. Relative changes (RC%) were calculated using the formula [(PGJ-PLA)/PLA × 100]. The normality of the data distribution was assessed using the Shapiro–Wilk test. A paired sample *T*-test was employed to ascertain the primary effect of the *T*-test on IFT performance. A two-way repeated measure ANOVA test (Condition × time) was utilized to determine the primary impact on VAFS and SLJ performance. Subsequently, Bonferroni’s *post hoc* test was conducted to identify pairwise differences. The analysis was carried out using SPSS (version 26, IBM-SPSS Inc., Chicago, IL, United States), with statistical significance considered at *p* ≤ 0.05. Also, the SWC was calculated as 0.2 times the within-subject SD in PLA (SWC = 0.2 × SD), following established methodologies for meaningful effect sizes in performance studies (38). Participants whose individual performance changes exceeded the SWC threshold in the PGJ conditions compared to PLA were classified as “responders.” The partial eta squared (η^2^) and Cohen’s d were computed as measures of effect size for both interaction effects and main effects. As per Cohen’s guidelines, a partial eta squared (η^2^) value of ≥ 0.01 represents a small effect, ≥ 0.059 indicates a medium effect, and ≥ 0.138 signifies a large effect (39). Cohen’s d was used to quantify the magnitude of pairwise differences, where values of 0.2, 0.5, and 0.8 correspond to small, medium, and large effects, respectively (40). These thresholds provide a framework for interpreting the practical significance of the observed.

## Results

**FIGURE 3:**
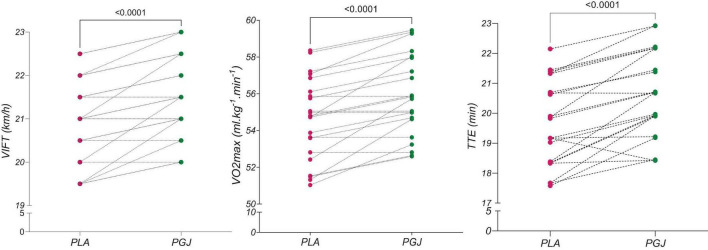
Individual responses in the placebo and purple grape juice conditions in the IFT test. VIFT, (the final) velocity–intermittent fitness test; VO_2max_, maximum oxygen consumption; TTE, time to exhaustion.

The descriptive characteristics of the measured variables in both conditions are presented in Table 2. The results of the paired *t*-test indicated an increase in VIFT [t = 5.66, *P* < 0.001, Cohen’s d = 0.58, 95% CI (0.36–0.78)], TTE [t = 5.89, *P* < 0.001, Cohen’s d = 0.69, 95% CI (37.8–79.1)], and VO_2*max*_ [t = 5.67, *P* < 0.001, Cohen’s d = 0.56, 95% CI (0.8–1.7)] following the consumption of PGJ compared to the placebo condition (Figure 3). Furthermore, the two-way repeated measure ANOVA revealed a main effect of time on SLJ performance (F = 82.2, *P* < 0.001, η^2^ = 0.8) and VAFS (F = 1094.6, *P* < 0.001, η^2^ = 0.98). However, the main effect of the condition (F = 3,5, *P* = 0.075, η^2^ = 0.14) and the interaction (condition × time) (F = 0.33, *P* = 0.71, η^2^ = 0.02) on SLJ performance were not found to be significant. Similarly, the main effect of the condition (F = 1.21, *P* = 0.28, η^2^ = 0.05) and the interaction (condition × time) (F = 2.67, *P* = 0.08, η^2^ = 0.11) on VAFS were also not significant.

**TABLE 2:** Descriptive characteristics (mean ± SDs) of measured variables in PGJ and placebo conditions.

Variable		PLA	PGJ	RC% (PGJ vs. PLA)
IFT parameters	VIFT (km/h)	20.90 ± 0.97	21.47 ± 0.98	↑ 2.7%
	VO_2max (ml/kg_^–1^_⋅*min*_^–1^_)_	54.47 ± 2.23	55.70 ± 2.21	↑ 2.3%
	TTE (min)	19.64 ± 1.40	20.61 ± 1.42	↑ 5.1%
SLJ (cm)	Baseline	263.68 ± 9.74	263.90 ± 9.61	↑ 0.1%
	0’-IFT	257.13 ± 9.50	257.45 ± 8.91	↑ 0.1%
	5’-IFT	260.86 ± 9.32	261.54 ± 9.51	↑ 0.3%
VAFS (mm)	Baseline	9.40 ± 3.52	8.18 ± 3.85	↓ 12.7%
	0’-IFT	95.04 ± 3.56	96.04 ± 3.68	↑ 1%
	5’-IFT	62.09 ± 10.51	59.77 ± 10.15	**↓** 3.7%

PGJ, purple grape juice, PLA, placebo, VAFS, visual analog fatigue scale, SLJ, standing long jump, IFT, 30-15 Intermittent Fitness Test; VIFT, (the final) velocity–intermittent fitness test; 0’-IFT, Immediately after IFT; 5’-IFT, 5 min after IFT; TTE, time to exhaustion, RC%, relative change.

**FIGURE 4:**
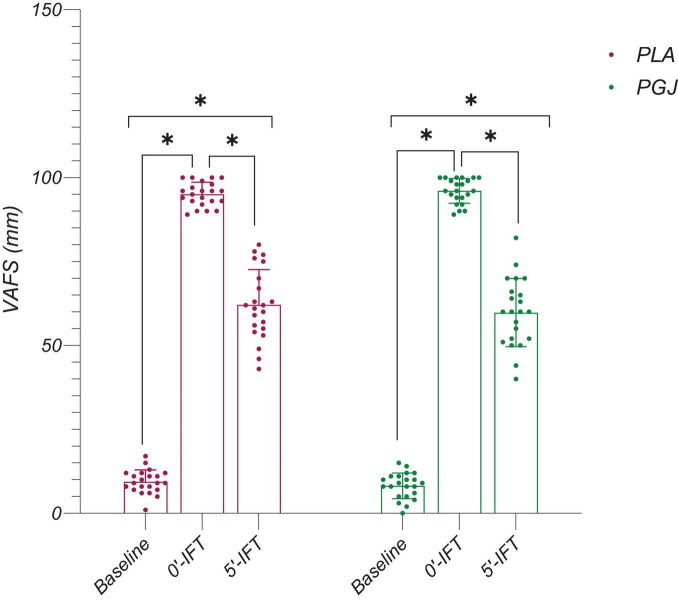
Mean and SDs and individual responses of perceived fatigue (VAFS) of participants in the placebo and purple grape juice conditions. *: Significant differences were observed between paired time points (*P* < 0.001).

**FIGURE 5:**
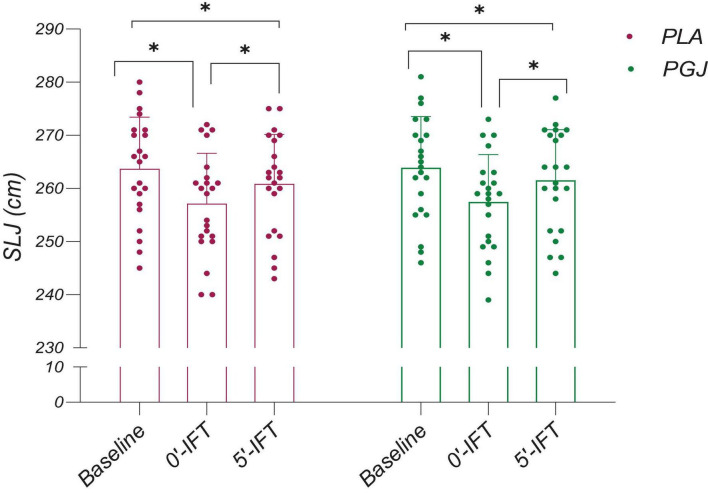
Mean and SDs and individual responses for the standing long jump (SLJ) performance of the placebo and purple grape juice conditions. *: Significant differences were observed between paired time points (*P* < 0.001).

The results of the multiple comparisons to assess the impact of time indicate that VAFS was significantly higher immediately after IFT (*P* < 0.001) and 5 min after IFT (*P* < 0.001) across all conditions, compared to the baseline (Figure 4). However, a significant reduction in VAFS was observed 5 min after IFT compared to immediately after IFT for PGJ and PLA conditions (*P* < 0.001) (Figure 4). Furthermore, SLJ performance for both conditions demonstrated a decrease from baseline to immediately after IFT (*P* < 0.001) and 5 min after IFT (*P* < 0.001) (Figure 5). Notably, SLJ performance showed considerable improvement 5 min after IFT compared to immediately after IFT for both conditions (PGJ&PLA) (*P* < 0.001) (Figure 5).

### Individual responses: smallest worthwhile change (SWC) analysis

The analysis of individual-level performance changes using the SWC approach provides detailed insights into the effectiveness of PGJ supplementation compared to the PLA condition. This analysis emphasizes the extent of response variability among participants across key performance indicators for IFT parameters, SLJ performance, and VAFS. Table 3 presents the analysis of individual-level changes using the SWC approach. The SWC analysis for 30-15 IFT parameters revealed improvements with PGJ supplementation compared to the PLA condition. For VIFT and VO_2*max*_, 77% of participants in the PGJ condition showed improvements exceeding the SWC threshold (Table 3). Additionally, regarding TTE, it was observed that 80% of the participants within the PGJ condition exhibited enhancements that exceeded the SWC threshold (Table 3).

**TABLE 3:** Individual-level changes based on e smallest worthwhile change (SWC) analysis across PGJ condition.

Variables		SWC (0.2 × SD_*PLA*_)	Responders PGJ (*n*)	Responders PGJ (%)
IFT parameters	VIFT (km/h)	0.19	17	77%
	VO_2max (ml/kg.BM)_	0.44	17	77%
	TTE (min)	0.28	20	90%
SLJ (cm)	Baseline	1.94	9	41%
	0’-IFT	1.9	10	45%
	5’-IFT	1.86	12	54%
VAFS (mm)	Baseline	0.70	12	54%
	0’-IFT	0.71	7	32%
	5’-IFT	2.1	13	59%

PGJ, purple grape juice; PLA, placebo; VAFS, visual analog fatigue scale; SLJ, standing long jump; IFT, 30-15 Intermittent Fitness Test; VIFT, (the final) velocity–Intermittent Fitness Test; 0’-IFT, Immediately after IFT; 5’-IFT, 5 min after IFT; TTE, time to exhaustion.

The SWC analysis for SLJ performance indicated improvements in partial participants with PGJ supplementation compared to the PLA condition. At baseline, 41% of participants in the PGJ condition exhibited above-threshold SWC relative to PLA. Immediately after IFT, this increased to 45%, and 5 min after IFT, it rose further to 54% (Table 3).

The SWC analysis for perceived fatigue (VAFS) revealed improvements in some participants with PGJ supplementation compared to the PLA condition. At baseline, 54% of participants reported perceived fatigue levels above the threshold for a SWC. After completing the IFT protocol, this percentage decreased to 32%, indicating an acute increase in fatigue. However, 5 min post-IFT, 59% of participants in the PGJ condition experienced perceived fatigue levels exceeding the SWC threshold, which was significantly higher than those in the PLA condition during the same time frame (Table 3).

## Discussion

The study presented novel findings on the effects of PGJ in elite male soccer players. The findings of this study demonstrate that consuming PGJ (10 ml/kg BM) significantly enhances specific endurance performance parameters, such as VIFT, TTE, and VO_2*max*_, in young male elite soccer players. However, no significant effects were observed on perceived fatigue or power performance, including SLJ. While individual-level analyses revealed some variability, a notable proportion of participants showed improvements in endurance-related parameters with purple grape juice supplementation compared to the placebo condition.

Until this study, the effects of PGJ on intermittent endurance performance (30-15 IFT) had not been investigated. However, de Lima Tavares Toscano et al. (25) reported that a single dose of PGJ (10 ml/kg BM) could increase the endurance of trained runners (TTE = 18.7%). In the present study, the increase in the TTE of PGJ compared to PLA was 5%. Although both studies show the ergogenic effects of PGJ on endurance performance, the inconsistency in the percentage of changes may be due to methodological differences between studies. For example, de Lima Tavares Toscano et al. (25) conducted their research using a running protocol that required participants to continue until exhaustion at 80% VO_2*max*_. In contrast, the present study implemented a 15-30 IFT approach. In addition, the present study’s sample size was larger than that of de Lima Tavares Toscano et al. (25) (*n* = 22 vs *n* = 14), which may have influenced the final results. It is important to highlight that the participants in the de Lima Tavares Toscano et al. (25) study were recreational runners, whereas the current study concentrated on elite soccer players. Therefore, the differences in training status and athlete levels may contribute to the observed variations in the results. In another study, Toscano et al. (26) showed that 28 days of grape juice supplementation in recreational runners significantly increased TTE (15.3%) and aerobic capacity (2.2%) compared to placebo. Although these findings are promising, human studies on the effects of natural grape juice on physical performance are still scarce. Therefore, more interventions are necessary to determine the effects of grape juice supplementation on athletes’ performance.

Some mechanisms could explain the ergogenic effects of grape juice on endurance performance. The improvement in endurance performance in the present study may be due to the carbohydrate content of PGJ compared to PLA. In the current study, participants in the PGJ condition consumed PGJ within 1–4 h before IFT, which contained ∼113 g of carbohydrates (∼1.6 g of CHO per kilogram of body mass) and 450 kcal. These values are consistent with the ACSM recommendations of consuming 1–4 g of carbohydrates/kg body mass, 1–4 h before exercise (41). In addition, there is evidence that consuming carbohydrate solutions before exercise increases the performance of high-intensity interval training (42). Carbohydrates consumed in meals and/or snacks within 1–4 h before exercise may help improve body glycogen stores, especially liver glycogen (43). In addition, it may be a source of gut glucose release during exercise (43). It can lead to glycogen sparing and improve performance in high-intensity intermittent exercise (44, 45). Muscle glycogen sparing can explain why players consuming carbohydrates can maintain high-intensity running during the second half of soccer matches. For example, it has been shown that when soccer players drank 400 ml of a carbohydrate solution (16% maltodextrin) before and during halftime, they covered 40% more distance in the second half of the game than a placebo (46). Furthermore, muscle glycogen depletion in subcellular glycogen compartments (e.g., sarcoplasmic reticulum) leads to a concomitant decrease in muscle calcium handling (47). On the other hand, decreasing the calcium release from the sarcoplasmic reticulum decreases the peak output power (48). Low muscle glycogen may therefore affect calcium flow and impairs muscle contractility (44). Therefore, the better performance of PGJ compared to placebo in IFT may be due to glycogen sparing and better muscle calcium flow. Evidence suggests that CHO may have a “non-metabolic” central effect (49, 50). According to the “central fatigue hypothesis,” the essential amino acid tryptophan, known as a precursor of serotonin, is implicated in central nervous system fatigue during prolonged exercise (51). It seems that CHO reduces the amount of tryptophan that crosses the blood-brain barrier, thereby reducing the concentration of serotonin in the brain (52). However, in the present study, there was no significant difference between placebo and PGJ in perceived fatigue at baseline, 0 and 5 min after IFT. In this study, participants performed IFT to exhaustion; it is reasonable to note that regardless of the placebo or PGJ condition, immediately after IFT, they were at their highest levels of perceived fatigue. This is why there is a significant difference in perceived fatigue in IFT. However, the increase in TTE after PGJ compared to placebo and the lack of significant difference in perceived fatigue suggest that PGJ effectively delayed fatigue. Measuring fatigue indices and perceived fatigue scales during a submaximal endurance test will reveal better the effects of PGJ on peripheral and central fatigue mechanisms. Therefore, it is suggested that, in addition to performance, metabolic and neurogenic aspects of PGG should also be addressed in future studies.

Regardless of carbohydrate content, some studies have shown that PGJ has increased endurance performance. For example, de Lima Tavares Toscano et al. (25) showed that a single dose of PGJ increased running time to exhaustion compared to a placebo with the same volume and CHO content (26). According to de Lima Tavares Toscano et al. (25), the antioxidant compounds in PGJ may delay the mechanism of muscle fatigue by reducing oxidative stress and thus lead to improved endurance performance (25). In addition, the polyphenols in grape juice have anti-inflammatory (21, 26, 28, 53) and vasodilating (54–57), properties that may improve muscle oxygen supply. However, further studies are needed to confirm these mechanisms or even determine other components that delay fatigue due to grape juice’s polyphenolic, anti-inflammatory, and vasodilatory properties.

Jumping ability is an essential aspect of soccer performance; however, there is limited evidence of pre/during exercise carbohydrate intake on jumping performance in soccer players. In the present study, grape juice supplementation did not significantly affect horizontal jumping ability at baseline, immediately after and 5 min after IFT. Consistent with these findings, Goulart et al. (28) showed that 14 days of grape juice supplementation had no effect on horizontal countermovement jump (28). This is the only study that directly examined the impact of grape juice on jumping performance. Therefore, due to the lack of evidence, it is suggested that in future studies, the effect of grape juice on anaerobic performance and power should be considered with different timing, dose, and study design. In addition, based on previous studies, the effect of carbohydrates on jumping performance is contradictory. For example, a study of recreational soccer players showed that consuming a carbohydrate drink (7.5%) before and during a specific soccer performance test had no effect on jumping ability compared to a placebo (58). Other evidence confirms the lack of improvement in jumping performance after consuming a carbohydrate drink (44, 59–61). Instead, Winnick et al. (62) showed that consuming a carbohydrate drink (6%) during an intense basketball distance test improved jumping performance in the fourth quarter (62). The findings of Winnick et al. (62) about jumping performance are inconsistent with the present study’s findings in 5 min after IFT. However, it should be noted that Winnick et al. (62) used the average of 20 vertical jumps as the final score. In contrast, the highest horizontal jump among the three attempts was recorded as the final score in the present study. In addition, the difference in the type of carbohydrates consumed (PGJ vs. 6% CHO beverage), the timing of supplementation (1–4 h before exercise vs. before and during exercise), and the training status of the subjects can contribute to the disparity of the findings.

The smallest worthwhile change (SWC) analysis is a powerful tool for assessing individual-level responses to interventions, particularly in sports nutrition and performance research (38, 39). It provides a precise threshold to identify meaningful changes in performance parameters, distinguishing actual improvements from natural variability (39). Recent studies have demonstrated the utility of SWC in evaluating natural supplements, such as polyphenol-rich grape juice, for their potential to enhance endurance, power, and recovery (25, 30). This approach allows researchers to go beyond group averages, highlighting individual variability in response to supplementation, which factors like baseline fitness, metabolism, and genetic predisposition may influence (38, 50). For example, SWC analysis has been applied to assess the effectiveness of carbohydrate-rich beverages in improving high-intensity performance, revealing significant individual differences in endurance and fatigue management (43, 45). By focusing on individualized responses, SWC provides a nuanced perspective on the ergogenic potential of nutritional interventions, emphasizing the importance of potential personalized strategies for optimizing athletic performance.

The results from the SWC analysis reveal substantial individual variability in the response to PGJ supplementation, particularly in parameters associated with aerobic endurance. Parameters such as VO_2*max*_, VIFT, and TTE showed meaningful improvements, with 77% of participants surpassing the SWC threshold for both VO_2*max*_ and VIFT. These findings suggest a robust ergogenic effect of PGJ in enhancing the ability to sustain high-intensity intermittent exercise. Comparable results have been observed in studies evaluating carbohydrate-based supplementation, where improved glycogen availability and delayed fatigue mechanisms were linked to performance gains (63). However, the variability observed in this study underscores the importance of considering individual factors, such as metabolic efficiency, baseline fitness, genotype, and habitual dietary patterns, in determining the effectiveness of nutritional interventions (29, 38) Based on these results, de Sousa et al. have revealed that the effect of purple grape juice on endurance performance is influenced by genetic factors (31). Consequently, future research must explore the influence of genetic factors on the efficacy of nutritional supplements. PGJ supplementation also influenced explosive power, as measured by the SLJ test. While group-level changes were insignificant, SWC analysis revealed that 41% of participants exceeded the meaningful change threshold at baseline, increasing to 45% post-IFT and 54% 5 min into recovery. This improvement in SLJ performance may be attributed to PGJ’s anti-fatigue and recovery-enhancing properties, likely driven by its polyphenol content, which has been shown to reduce oxidative stress and inflammation (25, 30). Additionally, the carbohydrate composition of PGJ may contribute to glycogen sparing during high-intensity exercise, facilitating better recovery and sustained power output (45, 63). These findings align with previous research on natural ergogenic aids, such as beetroot juice and tart cherry, which have demonstrated similar individual variability in enhancing endurance and recovery parameters (64–66). Notably, the proportion of responders in the PGJ group is consistent with studies evaluating other polyphenol-rich supplements, suggesting a comparable efficacy profile while offering the additional benefits of carbohydrate loading. The observed variability highlights the limitations of group-level analyses in sports nutrition research and the critical role of SWC analysis in identifying meaningful individual responses. While most participants exhibited significant improvements, a subset did not meet the SWC threshold, emphasizing the need for personalized nutritional strategies. Future studies should investigate this variability’s mechanisms, including genetic predisposition, gut microbiota composition, and enzymatic activity affecting polyphenol metabolism (66, 67). Moreover, exploring the dose-response relationship and long-term effects of PGJ supplementation may provide further insights into optimizing its use for athletic performance. In summary, PGJ supplementation significantly improved aerobic endurance and recovery parameters for many participants, particularly in high-intensity intermittent sports. These results and the potential benefits of reduced fatigue and enhanced recovery support its role as a promising natural ergogenic aid. However, the response variability reinforces the importance of individualizing supplementation protocols to maximize efficacy and ensure consistent benefits.

Despite the findings of this study, it is important to acknowledge several limitations. Firstly, the sample size of 22 elite young male soccer players, while more prominent than in some similar studies, may limit the generalizability of the results to a broader population of athletes in different sports. Future research should consider including a more diverse sample to enhance external validity. Additionally, the study only examined the acute effects of PGJ supplementation on specific endurance performance without assessing the long-term effects or adaptations that might occur with regular intake. Moreover, while the controlled diet and training conditions standardized macronutrient intake, they may not fully replicate real-world scenarios where dietary habits can vary significantly. The timing of PGJ ingestion (1–4 h pre-exercise) may also affect individual responses due to variations in metabolism and digestive efficiency among participants. Lastly, while valuable, the reliance on perceived fatigue measured by a visual analog scale may not encompass all dimensions of fatigue and could benefit from including additional physiological markers. Therefore, while the study offers insights into PGJ’s effects on endurance performance, the limitations warrant caution when interpreting the results and suggesting directions for future research. For example, a future study in women soccer players on the effects of purple grape juice on exercise performance is warranted.

## Conclusion

The study found that consuming 10 ml/kg of PGJ before high-intensity intermittent exercise significantly improved endurance parameters such as VO_2*max*_, VIFT, and TTE. However, no effect was observed on horizontal jump performance. Notably, 77% of participants exceeded the SWC threshold for VO_2*max*_ and VIFT, indicating a strong positive effect for most elite male football players, likely influenced by PGJ’s anti-fatigue properties and individual metabolic differences. These findings suggest PGJ may enhance endurance, mainly when consumed before training or matches. Therefore, our study not only reflects the combined polyphenol and carbohydrate effects of PGJ, but also supports the practicality of using natural beverages as pre-exercise strategies in elite athletes. However, further research is needed to clarify the mechanisms and optimize its benefits for high-intensity sports like soccer.

## Data Availability

The raw data supporting the conclusions of this article will be made available by the authors, without undue reservation.
